# Supplementary material for: Causal interplay between lactose intolerance and gut microbiota: a combined bidirectional Mendelian randomization and in vivo validation study
Source: Front Nutr. 2026 Jun 1;13:1803337. doi: 10.3389/fnut.2026.1803337 (PMC13265576; doi:10.3389/fnut.2026.1803337)
Supplement: Supplementary file 1 [file Data_Sheet_1.zip › supplementary materials/Forward/forest plot/ebi-a-GCST90027647.finngen_R12_E4_LACTONAS.pdf]

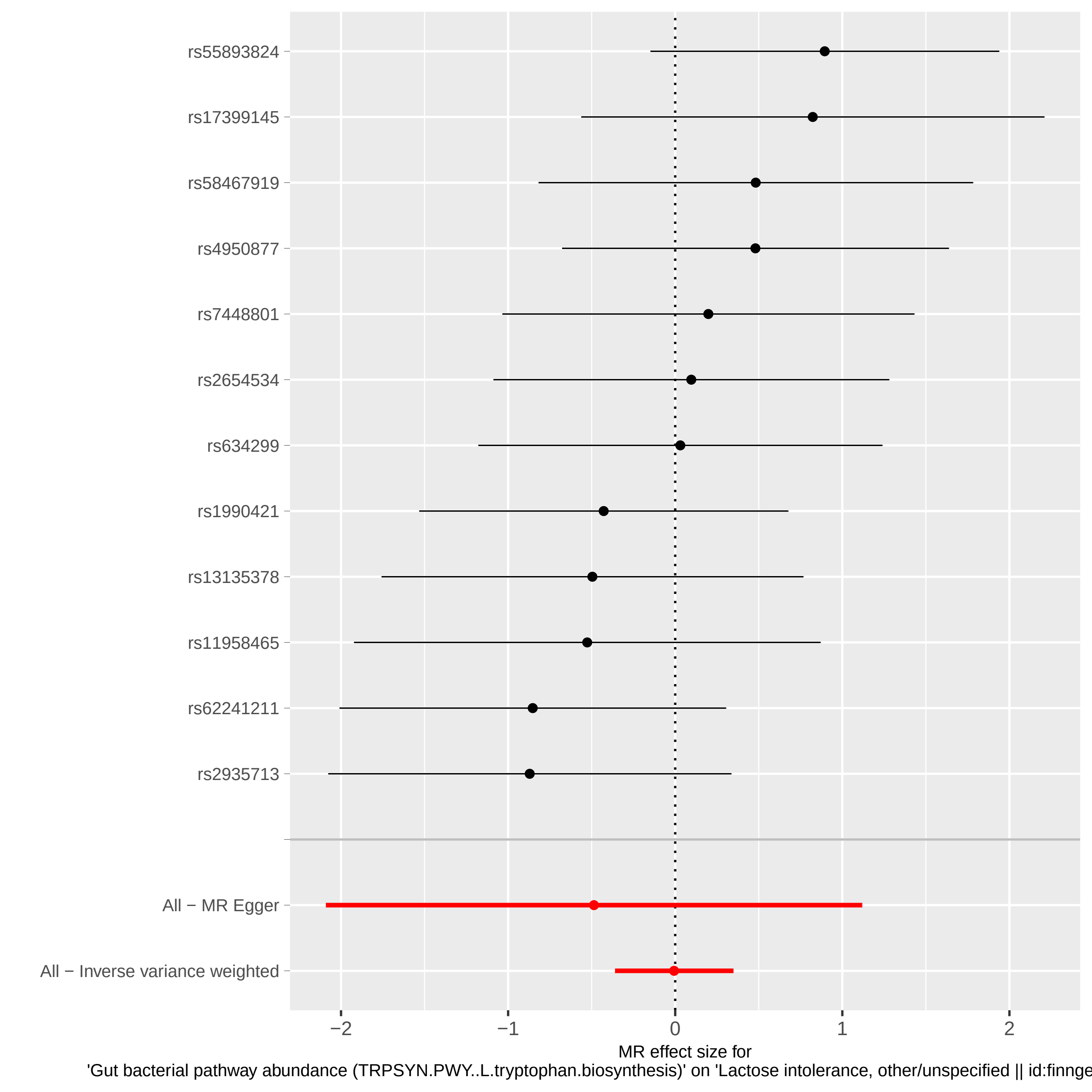
rs55893824
rs17399145
rs58467919
rs4950877
rs7448801
rs2654534
rs634299
rs1990421
rs13135378
rs11958465
rs62241211
rs2935713
All − MR Egger
All − Inverse variance weighted
−2
−1
0
1
2
MR effect size for
'Gut bacterial pathway abundance (TRPSYN.PWY..L.tryptophan.biosynthesis)' on 'Lactose intolerance, other/unspecified || id:finnge
